# Mine safety assessment based on basic event importance: grey relational analysis and bow tie model

**DOI:** 10.1098/rsos.180397

**Published:** 2018-08-08

**Authors:** Qingwei Xu, Kaili Xu, Li Li, Xiwen Yao

**Affiliations:** Key Laboratory of Ministry of Education on Safe Mining of Deep Metal Mines, School of Resources and Civil Engineering, Northeastern University, Shenyang 110819, People's Republic of China

**Keywords:** safety assessment, coal and gas outburst, fault tree analysis, importance, grey relational analysis, bow tie model

## Abstract

Safety assessments are a crucial first step in preventing coal and gas outburst accidents. The main purpose of this study was to create a new accident prevention technique using a novel safety assessment method based on fault tree basic event importance, grey relational analysis and the bow tie model. The innovation of the proposed method lies in generating the composite importance of a basic event from the fundamental importance via grey relational analysis; bow tie analysis serves to reveal the most critical basic event. First, the minimal cut sets and minimal path sets of a coal and gas outburst accident are determined by fault tree analysis. The role of minimal cut and path sets is determined and the coal and gas outburst occurrence frequency is calculated accordingly. Second, the structure, probability, critical and Fussell–Vesely importance ranked basic events differently due to different aspects of the basic events as investigated. We establish a composite importance to represent single basic events and achieved new ranking results by grey relational analysis. Third, the critical basic event low permeability coefficient is analysed via bow tie model and safety measures are defined which prevent the dangerous consequences of a low permeability coefficient. An actual coal and gas outburst accident is used as a case study to test the feasibility and effectiveness of the proposed method.

## 1. Introduction

Coal mining has been largely responsible for the dramatic uptick in economic and social development in China [1–3]. Coal has also accounted for about 60% of the country's total primary energy consumption in recent years [4,5]. Mining is dangerous and accidents are not altogether uncommon; coal and gas outbursts pose the most serious threat to the safety of the mine and miners [6–8]. Accurate safety assessments are a crucial first step in preventing coal and gas mine outburst accidents.

Fault tree analysis is the traditional safety assessment method [8,9]. The main goal of fault tree analysis is to explore the potential causes of system failure and predict the occurrence probability of a top event [10]. Fault tree analysis has been applied to mine safety [11,12], construction industry [13,14], gas transmission [15,16], communication and transportation [17,18], ecological system [19], environmental pollution [20] and electric power [21] applications. Zhang *et al*. [11] analysed transportation accidents in the surface mining field based on fault tree analysis. Chi *et al*. [13] applied fault tree analysis to investigate falling accidents in the construction industry. Dong & Yu [15] calculated the failure probability of gas transmission by fault tree analysis. Ding *et al*. [18] studied subway tunnel accidents using fault tree analysis. Kabir [22] made an overview of fault tree analysis per its limitations and various extensions.

After calculating the occurrence probability of a top event by fault tree analysis, it is necessary to determine the importance of basic events so as to identify appropriate targeted measures. Structure importance [23], probability importance [24,25], critical importance [26,27] and Fussell–Vesely importance [28,29] are typically used for this purpose. The importance ranking is generally inconsistent due to different aspects of basic events [24,26]. Joint importance was proposed by Hong & Lie [30] to calculate the importance of dual components, but there is as yet no reasonable or normative composite importance that fully encompasses a single basic event. There is demand for new techniques, to this effect, to calculate the composite importance of a single basic event.

Grey relational analysis is also frequently used for safety assessment; it is an important part of grey system theory [31]. Grey relational analysis serves to determine the order of indicators by calculating the grey relational degree among them [32]. It has been used in decision-making [33], green supplier selection [34] and energy consumption [35], among other applications. In this study, we took full advantage of the ranking ability of grey relational analysis to calculate the composite importance of a single basic event.

After determining the composite importance of basic events by grey relational analysis, corresponding measures should be identified to enhance safety within the mining enterprise. The bow tie model integrates basic causes, possible consequences and corresponding safety measures of an accident in a transparent diagram [36]. In this study, we used the bow tie model to analyse the critical events threatening safe production in the mine.

The purpose of this study was to build a novel safety assessment method for safe mine production. The innovation of the proposed method lies in generating the composite importance of a basic event from the fundamental importance via grey relational analysis, while bow tie analysis reveals the most critical basic event. We used grey relational analysis and the bow tie model as an extension of previous studies on fault tree analysis [11,12,26]. To take different aspects of a single basic event into consideration, we used a composite importance for the single basic event as calculated by grey relational analysis. The critical event can then be deeply analysed with the bow tie model to identify key safety measures.

The remainder of this paper is organized as follows. The fundamental theories relevant to the proposed method are summarized in §2. Our case study is presented in §3. The results are discussed in §4 and conclusions are provided in §5.

## 2. Methods

The theoretical basis of fault tree analysis, four types of importance, grey relational analysis and bow tie model as included in the proposed safety assessment method are discussed in this section.

### 2.1. Proposed method framework

**Figure 1.**
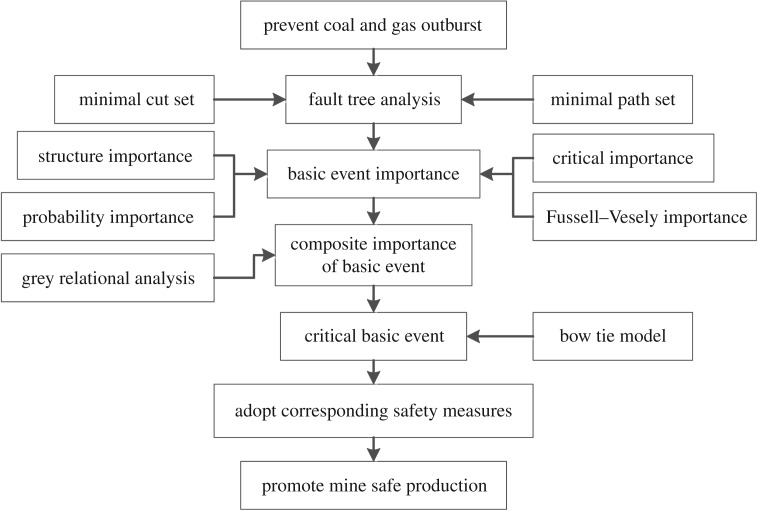
Framework of the proposed composite safety assessment method.

The framework of the proposed method is shown in figure 1.

As shown in figure 1, the first step of the proposed method is fault tree analysis of a coal and gas outburst to determine the role of the minimal cut and path sets. The composite importance of the basic event is then defined based on the fundamental importance by grey relational analysis. Third, the critical basic event of the coal and gas outburst is then analysed by bow tie model. Finally, corresponding safety measures which enhance mine safety are determined accordingly.

### 2.2. Fault tree analysis

Fault tree analysis is one of the most important safety system analysis methods available [8,9]. It works by building a logical relationship between possible accidents and causes. The occurrence probability of a top event can be calculated by the minimal cut set as follows:

2.1 $$P(T)=\sum_{r=1}^{k}\prod_{X_{i}\in E_{r}}q_{i}-\sum_{1\leq r<s\leq k}\prod_{X_{i}\in E_{r}\cup E_{s}}q_{i}+\cdots +{\left(-1\right)}^{k-1}\prod_{\underset{X_{i}\in E_{r}}{r=1}}^{k}q_{i},$$

where *E_r_* is the minimal cut set; *q_i_* is the occurrence probability of a basic event; *r*, *s* are the ordinal of the minimal cut set, *r* < *s*; *i* is the ordinal of the basic event, *X_i_* ∈ *E_r_*; *k* is the number of minimal cut sets; 1 ≤ *r *< *s *≤ *k* denotes a combined ordinal of the *r*th and *s*th minimal cut set; *X_i_* ∈ *E_r_* denotes the *i*th basic event which belongs to the *r*th minimal cut set; and *X_i_* ∈ *E_r_*∪*E_s_* denotes the *i*th basic event which belongs to the *r*th or *s*th minimal cut set.

If the minimal cut set value is large, it is difficult to calculate the occurrence probability of the top event by the minimal cut set. The occurrence probability of the top event can be calculated by the minimal path set:

2.2 $$P(T)=1-\sum_{r=1}^{l}\prod_{X_{i}\in P_{r}}\left(1-q_{i}\right)+\sum_{1\leq r<s\leq l}\prod_{X_{i}\in P_{r}\cup P_{s}}\left(1-q_{i}\right)-\cdots -{\left(-1\right)}^{l-1}\prod_{\underset{X_{i}\in P_{r}}{r=1}}^{l}\left(1-q_{i}\right),$$

where *P_r_* is the minimal path set; *r*, *s* are the ordinal of the minimal path set, *r* < *s*; *l* is the number of the minimal path set; 1 ≤ *r *< *s *≤ *l* denotes a combination ordinal of the *r*th and *s*th minimal path sets; *X_i_* ∈ *P_r_* denotes the *i*th basic event which belongs to the *r*th minimal path set; and *X_i_* ∈ *P_r_*∪*P_s_* denotes the *i*th basic event which belongs to the *r*th or *s*th minimal path set.

The occurrence probability of the basic event must be calculated before calculating the occurrence probability of the top event. If the object cannot be recovered, it can be calculated as follows:

2.3 $$q=1-{\text{e}}^{-\lambda t},$$

where *λ* is the object failure rate with exponential distribution and *t* is the running time of the object.

#### 2.2.1. Structure importance

Structure importance does not relate to the occurrence probability of basic events or any assumption that they are equal, but works simply to facilitate structural analysis of the influence of basic events on the top event [23]. Structure importance can be calculated as follows:

2.4 $$I_{S}(i)=\frac{1}{k}\sum_{r=1}^{k}\frac{1}{m_{r}},$$

where *m_r_* is the number of basic events of the *r*th minimal cut set.

#### 2.2.2. Probability importance

Probability importance is the degree to which the top event's occurrence probability influences the occurrence probability of the basic event [24,25]. Probability importance is also a type of Birnbaum importance which can be calculated as follows:

2.5 $$I_{B}(i)=\frac{\partial P(T)}{\partial q_{i}}.$$

#### 2.2.3. Critical importance

Critical importance is the rate of occurrence probability change in the top event caused by variations in the occurrence probability of the basic event [26,27]; it is calculated as follows:

2.6 $$I_{C}(i)=\underset{\Delta q_{i}\to 0}{\lim}\frac{\Delta P(T)/P(T)}{\Delta q_{i}/q_{i}}=\frac{q_{i}}{P(T)}\cdot I_{B}(i)$$

#### 2.2.4. Fussell–Vesely importance

Fussell–Vesely importance is the rate of change in top event occurrence probability caused by basic event non-occurrence [28,29], which can be calculated as follows:

2.7 $$I_{\mathrm{FV}}(i)=\frac{P(T)-P(T|q_{i}=0)}{P(T)}.$$

### 2.3. Grey relational analysis

Grey relational analysis is a branch of grey system theory wherein an optimal project can be identified by calculating the grey relational degree between the ideal project and projects [31,32]. The project is favourable when it has a relatively high grey relational degree. Grey relational analysis is less demanding in terms of the quantity and regularity of samples and can be easily operated. The computational process of grey relational analysis is as follows.

Let the data series of evaluation indicators be *A *= [*a_ij_*], where *a_ij_* is the original data of the *j*th evaluation indicator of the *i*th evaluation project, *m* the number of evaluation projects and *n* the number of evaluation indicators. The matrix *B *= [*b_j_*] is the ideal project, where *b_j_* is the ideal value of the *j*th evaluation indicator. For positive indicators, the ideal value is the maximum; for negative indicators, it is the minimum; for moderate indicators, it should be determined based on the physical truth.

If all the evaluation indicators of a project can reach the ideal value, the given project is certainly optimal. The evaluation indicators of a project do not reach the same, ideal values in reality, however. Grey relational analysis is applicable in such cases.

Let the ideal project *B* be the reference sequence and evaluation projects *A* be the sequences compared. The grey relational coefficient of the *j*th evaluation indicator of the *i*th evaluation can be calculated as follows:

2.8 $$\xi_{ij}=\frac{\underset{1\leq j\leq n}{\underset{1\leq i\leq m}{\min}}|b_{j}-a_{ij}|+\rho \underset{1\leq j\leq n}{\underset{1\leq i\leq m}{\max}}|b_{j}-a_{ij}|}{\left|b_{j}-a_{ij}|+\rho \underset{1\leq j\leq n}{\underset{1\leq i\leq m}{\max}}|b_{j}-a_{ij}\right|}.$$

To prevent data distortion due to the maximum absolute value and increase the significant difference between grey relational coefficients, the resolution coefficient *ρ*∈(0,1) is introduced in formula (2.8) (usually *ρ* = 0.5). Let the evaluation indicators weights be *W *= [*w*_1_, *w*_2_,…, *w_n_*], then the grey relational degree of evaluation projects can be determined as follows:

2.9 $$r_{i}=\sum_{j=1}^{n}\xi_{ij}\times w_{j},\;i=1,2,\ldots ,m$$

The larger the grey relational degree, the closer the evaluation project is to the ideal project. The order of the evaluation projects can be determined through this process to successfully identify the optimal project.

### 2.4. Bow tie model

**Figure 2.**
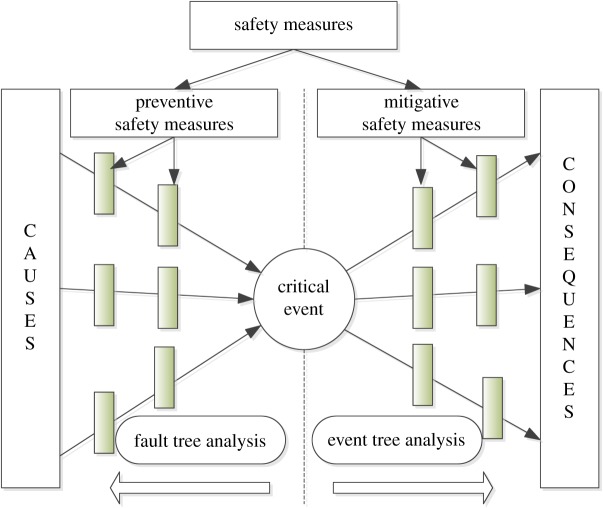
Bow tie model sketch.

The bow tie model (figure 2) consists of fault tree analysis on the left and event tree analysis on the right [36]. The critical event is centred in the bow tie; on the left are the basic events that may result in the critical event and on the right are the consequences of the critical event. Safety measures are necessary to prevent the critical event. Preventive safety measures are set on the left to prevent the causes of the critical event, and mitigative safety measures are set on the right which reduce the consequences of the critical event.

## 3. Results

In the underground mining process, fragmentized coal and rock as well as gas are suddenly thrown into the mining space from the coal or rock body due to the combined action of crustal stress and gas pressure. This anomalous, dynamic phenomenon is a coal and gas outburst. Outbursts happen quickly, over the course of several seconds or potentially minutes. It is critically important to identify the major determining factors of an outburst and employ targeted prevention measures to ensure safe production in the mine.

**Figure 3.**
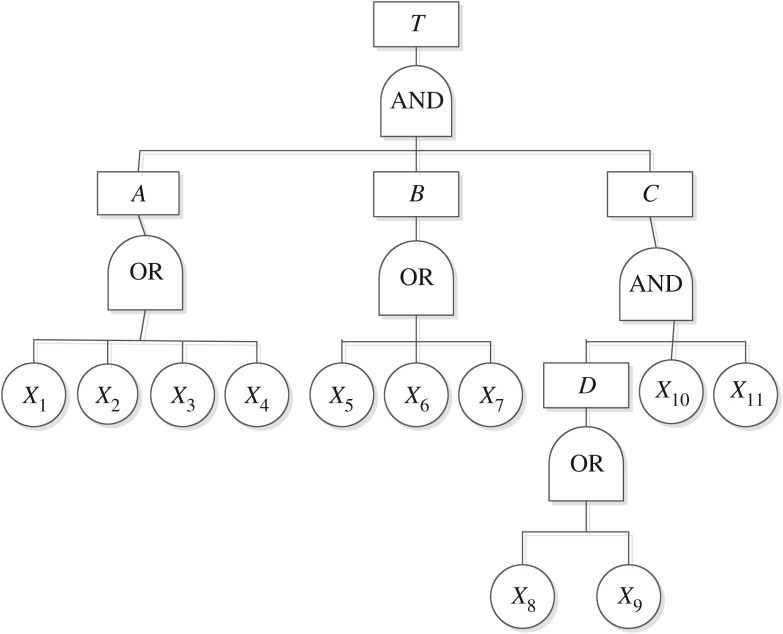
Fault tree of coal and gas outburst.

The combined effects hypothesis regards a coal and gas outburst as the result of a combination of crustal stress, gas and the physical mechanics of coal. The combined effects hypothesis has been well established within the scientific community [26]. A fault tree of the coal and gas outburst based on the combined effects hypothesis in *GC* coal mine is shown in figure 3 [37].

In figure 3, *T* denotes the coal and gas outburst; *A* denotes gas; *B* denotes crustal stress; *C* denotes the physical mechanics of coal; *D* denotes coal seam complexity; *X*_1_ denotes the coal seam gas pressure being equal to or greater than 0.74 MPa; *X*_2_ denotes coal seam gas content equal to or greater than 8 m^3^ t^−1^; *X*_3_ denotes the initial velocity of diffusion of coal gas being equal to or greater than 10 ml s^−1^; *X*_4_ denotes the maximum initial velocity of gas emission being equal to or greater than 5 l min^−1^; *X*_5_ denotes the variation of geologic structure; *X*_6_ denotes the stress concentration; *X*_7_ denotes the gas permeability of surrounding rock; *X*_8_ denotes the variations of thickness in the coal seam; *X*_9_ denotes coal seam dip angle variations; *X*_10_ denotes coal mass strength less than 0.5 MPa; *X*_11_ denotes permeability coefficient less than 0.1 m^2^/(MPa^2^ d).

The occurrence probability and importance of basic events are shown in table 1 [37].

**Table 1.** Occurrence probability and importance of basic events.

			importance ranking				
basic event	frequency/year	probability	*I*_S_	*I*_B_	*I*_C_	*I*_FV_	*I*_CI_
*x*_1_	0.5	0.3934	8	11	10	10	11
*x*_2_	0.833	0.5652	8	9	8	8	9
*x*_3_	1	0.6321	8	8	7	7	7
*x*_4_	0.833	0.5652	8	9	8	8	9
*x*_5_	0.15	0.1393	5	6	6	6	6
*x*_6_	0.05	0.0488	5	7	11	10	8
*x*_7_	0.833	0.5652	5	5	3	3	5
*x*_8_	0.167	0.1538	3	1	4	4	3
*x*_9_	0.1	0.0952	3	2	5	5	4
*x*_10_	1	0.6321	1	3	1	1	1
*x*_11_	1	0.6321	1	3	1	1	1

The occurrence probability of basic events was determined based on formula (2.3) as shown in table 1. The minimal cut set of the coal and gas outburst can be achieved based on the fault tree as follows:

3.1 T=A⋅B⋅C.

The total number of minimal cut sets was 24 after unfolding according to Boolean algebra: *E*_1 _= {*X*_1_, *X*_5_, *X*_8_, *X*_10_, *X*_11_}; *E*_2 _= {*X*_2_, *X*_5_, *X*_8_, *X*_10_, *X*_11_}; *E*_3 _= {*X*_3_, *X*_5_, *X*_8_, *X*_10_, *X*_11_}; *E*_4 _= {*X*_4_, *X*_5_, *X*_8_, *X*_10_, *X*_11_}; *E*_5 _= {*X*_1_, *X*_6_, *X*_8_, *X*_10_, *X*_11_}; *E*_6 _= {*X*_2_, *X*_6_, *X*_8_, *X*_10_, *X*_11_}; *E*_7 _= {*X*_3_, *X*_6_, *X*_8_, *X*_10_, *X*_11_}; *E*_8 _= {*X*_4_, *X*_6_, *X*_8_, *X*_10_, *X*_11_}; *E*_9 _= {*X*_1_, *X*_7_, *X*_8_, *X*_10_, *X*_11_}; *E*_10 _= {*X*_2_, *X*_7_, *X*_8_, *X*_10_, *X*_11_}; *E*_11 _= {*X*_3_, *X*_7_, *X*_8_, *X*_10_, *X*_11_}; *E*_12 _= {*X*_4_, *X*_7_, *X*_8_, *X*_10_, *X*_11_}; *E*_13 _= {*X*_1_, *X*_5_, *X*_9_, *X*_10_, *X*_11_}; *E*_14 _= {*X*_2_, *X*_5_, *X*_9_, *X*_10_, *X*_11_}; *E*_15 _= {*X*_3_, *X*_5_, *X*_9_, *X*_10_, *X*_11_}; *E*_16 _= {*X*_4_, *X*_5_, *X*_9_, *X*_10_, *X*_11_}; *E*_17 _= {*X*_1_, *X*_6_, *X*_9_, *X*_10_, *X*_11_}; *E*_18 _= {*X*_2_, *X*_6_, *X*_9_, *X*_10_, *X*_11_}; *E*_19 _= {*X*_3_, *X*_6_, *X*_9_, *X*_10_, *X*_11_}; *E*_20 _= {*X*_4_, *X*_6_, *X*_9_, *X*_10_, *X*_11_}; *E*_21 _= {*X*_1_, *X*_7_, *X*_9_, *X*_10_, *X*_11_}; *E*_22 _= {*X*_2_, *X*_7_, *X*_9_, *X*_10_, *X*_11_}; *E*_23 _= {*X*_3_, *X*_7_, *X*_9_, *X*_10_, *X*_11_}; *E*_24 _= {*X*_4_, *X*_7_, *X*_9_, *X*_10_, *X*_11_}.

We determined the minimal path set of the coal and gas outburst based on formula (3.1) using Boolean algebra: *P*_1 _= {*X*_1_, *X*_2_, *X*_3_, *X*_4_}; *P*_2 _= {*X*_5_, *X*_6_, *X*_7_}; *P*_3 _= {*X*_8_, *X*_9_}; *P*_4 _= {*X*_10_}; *P*_5 _= {*X*_11_}.

There were 24 minimal cut sets and five minimal path sets of this fault tree, which made it easy to calculate outburst occurrence probability using the minimal path set by formula (2.2); the result was *P*(*T*) = 0.0578. The outburst occurrence frequency was determined to be 0.0595 per year through transition by formula (2.3).

The structure importance of basic event *X*_1_ can be calculated by formula (2.4) as follows:

$$I_{S}(1)=\frac{1}{24}\cdot \left(\frac{1}{5}\cdot 6\right)=\frac{1}{20}.$$

The structure importance of other basic events is discussed in the electronic supplementary material. The structure importance ranking we obtained is shown in table 1.

The probability importance of basic event *X*_1_ can be calculated as follows:

$$I_{B}(1)=\frac{\partial P(T)}{\partial q_{1}}=0.0042.$$

The probability importance of other basic events is provided in the electronic supplementary material; the resulting ranking is shown in table 1.

The critical importance of basic event *X*_1_ can be calculated as follows:

$$I_{C}(1)=\frac{q_{1}}{P(T)}\cdot I_{B}(1)=0.0286.$$

The critical importance of other basic events is again provided in the electronic supplementary material; our ranking is shown in table 1.

The Fussell–Vesely importance of basic event *X*_1_ can be calculated as follows:

$$I_{\mathrm{FV}}(1)=\frac{P(T)-P(T|q_{1}=0)}{P(T)}=0.0294.$$

The Fussell–Vesely importance of other basic events is also provided in the electronic supplementary material and our ranking is shown in table 1.

## 4. Discussion

### 4.1. Discussion of fault tree analysis

The minimal cut set plays a very important role in accident analysis. It is indicative of risk in the system under analysis. There was a total of 24 minimal cut sets in our fault tree, indicating that a coal and gas outburst can be caused by 24 paths—i.e. that this coal mine is very dangerous. The minimal cut set also indicates the causes of a coal and gas outburst accident. The occurrence of a coal and gas outburst accident must involve the simultaneous occurrence of the basic events in one or several minimal cut sets. Once the coal and gas outburst accident has occurred, the causes of the accident can be quickly identified. The minimal cut set also reveals control direction and prevention measures to reduce the risk of an outburst; it makes clear the most dangerous accident mode and indicates potential opportunities to reduce the outburst occurrence probability. The occurrence probability of an outburst accident can be calculated by formula (2.1) based on the minimal cut set.

The minimal path set plays as important a role in accident analysis as the minimal cut set. First, it indicates the safety of the system. A coal and gas outburst accident can be prevented by preventing all the basic events in a random minimal path set. The best solution to ensure system safety can also be selected based on the minimal path set. Each minimal path set is a solution to preventing a gas outburst—the best solution must also be considered in regard to the necessary technology, time and money. The outburst occurrence probability can be calculated by formula (2.2) based on the minimal path set.

### 4.2. Composite importance based on grey relational analysis

As shown in table 1, the importance rankings we obtained were inconsistent due to differences in the aspects of the basic event we investigated. We sought a novel approach to taking different levels of importance into consideration. Grey relational analysis can be used to evaluate the sequence of different indicators by calculating the grey relational degree, so we used it to calculate the composite importance of basic events. The data matrix of varying importance is shown below.

$$A={\left[\begin{array}{ccccccccccc} 8 & 8 & 8 & 8 & 5 & 5 & 5 & 3 & 3 & 1 & 1\\ 10 & 8 & 7 & 8 & 6 & 11 & 3 & 4 & 5 & 1 & 1\\ 11 & 9 & 8 & 9 & 6 & 7 & 5 & 1 & 2 & 3 & 3\\ 10 & 8 & 7 & 8 & 6 & 10 & 3 & 4 & 5 & 1 & 1 \end{array}\right]}^{\prime }.$$

The ideal project composed of basic events was *B *= [1, 1, 1, 1]. The grey relational coefficient of basic event *X*_1_ was obtained as follows:

$$\xi_{1}=[\begin{array}{cccc} 0.4167 & 0.3571 & 0.3333 & 0.3571 \end{array}].$$

When four disparate types of importance are examined from different directions, it is difficult to distinguish which is more important. To resolve this issue, we assigned them all the same weight, that is, *W* = [0.25 0.25 0.25 0.25]′. The composite importance of basic event *X*_1_ can be calculated based on the grey relational coefficient and weights:

$$I_{\mathrm{CI}}(\text{1})=r_{1}=[\begin{array}{cccc} 0.4167 & 0.3571 & 0.3333 & 0.3571 \end{array}]\cdot {\left[\begin{array}{cccc} 0.25 & 0.25 & 0.25 & 0.25 \end{array}\right]}^{\prime }=0.\text{3661}.$$

The composite importance of other basic events is provided in the electronic supplementary material; our composite importance ranking is shown in table 1. Basic events *X*_10_, *X*_11_ were found to contribute the most to the occurrence of coal and gas outburst accidents.

### 4.3. Bow tie analysis of basic event *X*_11_

**Figure 4.**
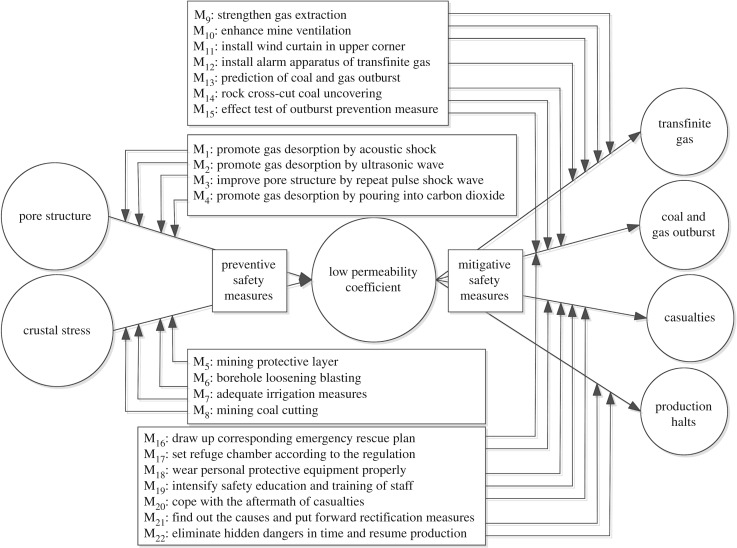
Bow tie analysis of low permeability coefficient.

The permeability coefficient reflects the complexity of gas flow in the coal seam, which is an important parameter to determine the feasibility of gas extraction. In previous studies [11,12,26], relatively simple analyses were conducted to identify critical factors in mine safety evaluations; arguably, they failed to identify the causes and consequences of critical factors and could not improve mine safe production effectively in practice. The occurrence probability of a low permeability coefficient was the largest among the basic events, so we selected it as the critical factor for bow tie analysis. We then identified the causes and consequences of a low permeability coefficient and identified corresponding measures to prevent accidents (figure 4).

The left of the bow tie is a fault tree analysis that includes two causes of the low permeability coefficient. The right of the bow tie is an event tree analysis that includes four consequences of the low permeability coefficient (figure 4). The risk of low permeability coefficient can be significantly reduced per the results of the bow tie analysis.

### 4.4. Results summary

We successfully determined the composite importance of a single basic event as calculated by grey relational analysis, then analysed the critical event via bow tie model using the proposed method. Our results confirm that this method can be successfully applied to evaluate mine safety. The primary advantages of our method are twofold. First, the composite importance of various aspects of a single basic event, which is defined based on grey relational analysis, effectively gives priority to the factors most likely to cause an outburst accident. Second, the critical basic event's low permeability coefficient was analysed via bow tie model for the first time in this study. This analysis reveals the causes and consequences corresponding to safety measures that may be adopted to prevent outbursts.

The structure [23], probability [24,25], critical [26,27] and Fussell–Vesely [28,29] importance of basic events are not consistent in the fault tree analysis of coal and gas outbursts due to differences in the basic events under analysis [24,26]. We established a composite importance that takes various aspects of single basic events into consideration based on grey relational analysis [31,32]. The basic events that contribute the most to the occurrence of a coal and gas outburst accident were determined to be low permeability coefficient and coal mass strength—these factors must be carefully regulated to safeguard the mine. Grey relational analysis is fundamentally clear and intelligible, and using it to integrate different aspects of importance effectively encompasses the significant qualities of basic events.

Traditional mine safety assessments based on fault tree analysis have afforded only a simple analysis of the critical basic event [11,12,26]; they do not effectively reveal the causes and consequences which are necessary to improve mine safe production. Here, we analysed the critical basic event low permeability coefficient with the bow tie model [36] to identify said causes and determine safety measures accordingly. Eight preventive safety measures were set on the left which halt the causes of a low permeability coefficient and 14 mitigative safety measures were set on the right which reduce the consequences of the low permeability coefficient. This type of bow tie analysis can significantly reduce the risk of coal and gas outbursts when applied correctly. It can also be extended to safety assessment in other industries.

To simplify the discussion, only the critical basic event low permeability coefficient was analysed via bow tie model in the present study. In future research, we plan to assess more critical basic events similarly to reveal new factors involved in safe mine production.

## 5. Conclusion

This paper proposed a novel safety assessment method based on importance as defined by grey relational analysis and bow tie model. Our main conclusions can be summarized as follows.

We identified 24 minimal cut sets and five minimal path sets of the coal and gas outburst accident by fault tree analysis; occurrence frequency of coal and gas outburst was calculated to be 0.0595 per year.

The structure, probability, critical and Fussell–Vesely importance are ranked differently due to different aspects of the basic events investigated. A composite importance of a single basic event was established as calculated by grey relational analysis which takes different aspects of the single basic event into consideration. The composite importance ranking result was *X*_10 _= *X*_11_ > *X*_8_ > *X*_9_ > *X*_7_ > *X*_5_ > *X*_6_ > *X*_3_ > *X*_2 _= *X*_4_ > *X*_1_, indicating that basic events *X*_10_, *X*_11_ contribute the most to coal and gas outbursts. To this effect, they must be the focus of safety measures taken in the mine to prevent accidents.

The critical basic event low permeability coefficient was analysed via bow tie model for the first time in this study. Eight preventive safety measures were set on the left which may halt the causes of a low permeability coefficient and 14 mitigative safety measures were set on the right which may reduce the consequences of the low permeability coefficient. The risk of outbursts due to a low permeability coefficient can be significantly reduced by bow tie analysis.

## Supplementary Material

Basic event importance

## Data accessibility

The importance of the basic events that are included in this paper has been uploaded as the electronic supplementary material.
