# Supplementary material for: Mine safety assessment based on basic event importance: grey relational analysis and bow tie model
Source: R Soc Open Sci. 2018 Aug 8;5(8):180397. doi: 10.1098/rsos.180397 (PMC6124069; doi:10.1098/rsos.180397)
Supplement: Basic event importance [file rsos180397supp1.doc]

***Royal Society Open Science***

Mine safety assessment based on basic event importance: gray relational analysis and bow tie model

**Qingwei Xu, Kaili Xu^*^, Li Li and Xiwen Yao**

Key Laboratory of Ministry of Education on Safe Mining of Deep Metal Mines, School of Resources and Civil Engineering, Northeastern University, Shenyang 110819, China

*Correspondence author: [xklsafety@163.com](mailto:xklsafety@163.com)

The structure importance of basic event can be calculated by Formula (4) as follows:

$$I_{S}\left(1\right)=\frac{1}{24}\cdot\left(\frac{1}{5}\cdot6\right)=\frac{1}{20}$$

$$I_{S}\left(2\right)=\frac{1}{24}\cdot\left(\frac{1}{5}\cdot6\right)=\frac{1}{20}$$

$$I_{S}\left(3\right)=\frac{1}{24}\cdot\left(\frac{1}{5}\cdot6\right)=\frac{1}{20}$$

$$I_{S}\left(4\right)=\frac{1}{24}\cdot\left(\frac{1}{5}\cdot6\right)=\frac{1}{20}$$

$$I_{S}\left(5\right)=\frac{1}{24}\cdot\left(\frac{1}{5}\cdot8\right)=\frac{1}{15}$$

$$I_{S}\left(6\right)=\frac{1}{24}\cdot\left(\frac{1}{5}\cdot8\right)=\frac{1}{15}$$

$$I_{S}\left(7\right)=\frac{1}{24}\cdot\left(\frac{1}{5}\cdot8\right)=\frac{1}{15}$$

$$I_{S}\left(8\right)=\frac{1}{24}\cdot\left(\frac{1}{5}\cdot12\right)=\frac{1}{10}$$

$$I_{S}\left(9\right)=\frac{1}{24}\cdot\left(\frac{1}{5}\cdot12\right)=\frac{1}{10}$$

$$I_{S}\left(10\right)=\frac{1}{24}\cdot\left(\frac{1}{5}\cdot24\right)=\frac{1}{5}$$

$$I_{S}\left(11\right)=\frac{1}{24}\cdot\left(\frac{1}{5}\cdot24\right)=\frac{1}{5}$$

The probability importance of basic event *X_1_* can be calculated by Formula (5) as follows:

$$I_{B}\left(1\right)=\frac{\partial P\left(T\right)}{\partial q_{1}}=0.0042$$

$$I_{B}\left(2\right)=\frac{\partial P\left(T\right)}{\partial q_{2}}=0.0059$$

$$I_{B}\left(3\right)=\frac{\partial P\left(T\right)}{\partial q_{3}}=0.0069$$

$$I_{B}\left(4\right)=\frac{\partial P\left(T\right)}{\partial q_{4}}=0.0059$$

$$I_{B}\left(5\right)=\frac{\partial P\left(T\right)}{\partial q_{5}}=0.0371$$

$$I_{B}\left(6\right)=\frac{\partial P\left(T\right)}{\partial q_{6}}=0.0336$$

$$I_{B}\left(7\right)=\frac{\partial P\left(T\right)}{\partial q_{7}}=0.0734$$

$$I_{B}\left(8\right)=\frac{\partial P\left(T\right)}{\partial q_{8}}=0.2230$$

$$I_{B}\left(9\right)=\frac{\partial P\left(T\right)}{\partial q_{9}}=0.2086$$

$$I_{B}\left(10\right)=\frac{\partial P\left(T\right)}{\partial q_{10}}=0.0914$$

$$I_{B}\left(11\right)=\frac{\partial P\left(T\right)}{\partial q_{11}}=0.0914$$

The critical importance of basic event *X_1_* can be calculated by Formula (6) as follows:

$$I_{C}\left(1\right)=\frac{q_{1}}{P\left(T\right)}\cdot I_{B}\left(1\right)=0.0286$$

$$I_{C}\left(2\right)=\frac{q_{2}}{P\left(T\right)}\cdot I_{B}\left(2\right)=0.0573$$

$$I_{C}\left(3\right)=\frac{q_{3}}{P\left(T\right)}\cdot I_{B}\left(3\right)=0.0757$$

$$I_{C}\left(4\right)=\frac{q_{4}}{P\left(T\right)}\cdot I_{B}\left(4\right)=0.0573$$

$$I_{C}\left(5\right)=\frac{q_{5}}{P\left(T\right)}\cdot I_{B}\left(5\right)=0.0895$$

$$I_{C}\left(6\right)=\frac{q_{6}}{P\left(T\right)}\cdot I_{B}\left(6\right)=0.0284$$

$$I_{C}\left(7\right)=\frac{q_{7}}{P\left(T\right)}\cdot I_{B}\left(7\right)=0.7185$$

$$I_{C}\left(8\right)=\frac{q_{8}}{P\left(T\right)}\cdot I_{B}\left(8\right)=0.5938$$

$$I_{C}\left(9\right)=\frac{q_{9}}{P\left(T\right)}\cdot I_{B}\left(9\right)=0.3437$$

$$I_{C}\left(10\right)=\frac{q_{10}}{P\left(T\right)}\cdot I_{B}\left(10\right)=1$$

$$I_{C}\left(11\right)=\frac{q_{11}}{P\left(T\right)}\cdot I_{B}\left(11\right)=1$$

The Fussell-Vesely importance of basic event can be calculated by Formula (7) as follows:

$$I_{FV}\left(1\right)=\frac{P\left(T\right)-P\left(T|q_{1}=0\right)}{P\left(T\right)}=0.0294$$

$$I_{FV}\left(2\right)=\frac{P\left(T\right)-P\left(T|q_{2}=0\right)}{P\left(T\right)}=0.0571$$

$$I_{FV}\left(3\right)=\frac{P\left(T\right)-P\left(T|q_{3}=0\right)}{P\left(T\right)}=0.0761$$

$$I_{FV}\left(4\right)=\frac{P\left(T\right)-P\left(T|q_{4}=0\right)}{P\left(T\right)}=0.0571$$

$$I_{FV}\left(5\right)=\frac{P\left(T\right)-P\left(T|q_{5}=0\right)}{P\left(T\right)}=0.0900$$

$$I_{FV}\left(6\right)=\frac{P\left(T\right)-P\left(T|q_{6}=0\right)}{P\left(T\right)}=0.0294$$

$$I_{FV}\left(7\right)=\frac{P\left(T\right)-P\left(T|q_{7}=0\right)}{P\left(T\right)}=0.7180$$

$$I_{FV}\left(8\right)=\frac{P\left(T\right)-P\left(T|q_{8}=0\right)}{P\left(T\right)}=0.5934$$

$$I_{FV}\left(9\right)=\frac{P\left(T\right)-P\left(T|q_{9}=0\right)}{P\left(T\right)}=0.3443$$

$$I_{FV}\left(10\right)=\frac{P\left(T\right)-P\left(T|q_{10}=0\right)}{P\left(T\right)}=1$$

$$I_{FV}\left(11\right)=\frac{P\left(T\right)-P\left(T|q_{11}=0\right)}{P\left(T\right)}=1$$

The composite importance of basic event can be calculated based on the gray relational coefficient and weights as follows:

$$I_{CI}\left(1\right)=r_{1}=\left[0.\text{4167 }0.\text{3571 }0.\text{3333 }0.3571\right]\cdot\left[0.\text{25 }0.\text{25 }0.\text{25 }0.25\right]'=0.3661$$

$$I_{CI}\left(2\right)=r_{2}=\left[0.\text{4167 }0.\text{4167 }0.\text{3846 }0.4167\right]\cdot\left[0.\text{25 }0.\text{25 }0.\text{25 }0.25\right]'=0.4087$$

$$I_{CI}\left(3\right)=r_{3}=\left[0.\text{4167 }0.\text{4545 }0.\text{4167 }0.4545\right]\cdot\left[0.\text{25 }0.\text{25 }0.\text{25 }0.25\right]'=0.4356$$

$$I_{CI}\left(4\right)=r_{4}=\left[0.\text{4167 }0.\text{4167 }0.\text{3846 }0.4167\right]\cdot\left[0.\text{25 }0.\text{25 }0.\text{25 }0.25\right]'=0.4087$$

$$I_{CI}\left(5\right)=r_{5}=\left[0.\text{5556 }0.\text{5000 }0.\text{5000 }0.5000\right]\cdot\left[0.\text{25 }0.\text{25 }0.\text{25 }0.25\right]'=0.5139$$

$$I_{CI}\left(6\right)=r_{6}=\left[0.\text{5556 }0.\text{3333 }0.\text{4545 }0.3571\right]\cdot\left[0.\text{25 }0.\text{25 }0.\text{25 }0.25\right]'=0.4251$$

$$I_{CI}\left(7\right)=r_{7}=\left[0.\text{5556 }0.\text{7143 }0.\text{5556 }0.7143\right]\cdot\left[0.\text{25 }0.\text{25 }0.\text{25 }0.25\right]'=0.6349$$

$$I_{CI}\left(8\right)=r_{8}=\left[0.\text{7143 }0.\text{6250 }1.0000\text{ }0.6250\right]\cdot\left[0.\text{25 }0.\text{25 }0.\text{25 }0.25\right]'=0.7411$$

$$I_{CI}\left(9\right)=r_{9}=\left[0.\text{7143 }0.\text{5556 }0.8333\text{ }0.5556\right]\cdot\left[0.\text{25 }0.\text{25 }0.\text{25 }0.25\right]'=0.6647$$

$$I_{CI}\left(10\right)=r_{10}=\left[1.0000\text{ }1.0000\text{ }0.7143\text{ }1.0000\right]\cdot\left[0.\text{25 }0.\text{25 }0.\text{25 }0.25\right]'=0.9286$$

$$I_{CI}\left(11\right)=r_{11}=\left[1.0000\text{ }1.0000\text{ }0.7143\text{ }1.0000\right]\cdot\left[0.\text{25 }0.\text{25 }0.\text{25 }0.25\right]'=0.9286$$
